# Changes in the University of Maryland

**Published:** 1893-10

**Authors:** 


### Editorial, Etc.

CHANGES IN THE UNIVERSITY OF MARYLAND.—The northeast corner of Greene and Lombard streets, hitherto a gloomy, walled-in spot, is now one of the handsomest corners in the city. The brick walls which hid the classic old building of the Maryland University Medical School and also portions of the Dental and Law Buildings have been taken away. The grounds have been graded and sodded, and edged with a granite curbing. The Medical building, which is of pure Greek architecture, with the lines of the Pantheon at Rome, the triangle panel and graceful columns, has been painted a light drab, and the columns look fresh and clean in white paint. The charter for the old building was granted by the legislature in 1808. The money was raised by a lottery, in charge of John Eager Howard, James McHenry, James Calhoun, Charles Ridgely of Hampden, Wm. Gwynn, Solomon Birckhead and others who obtained $40,000.

The ground was bought from the old Howard estate, and the building was begun May 7, 1812. The present improvements also include interior repainting and changes in the location of all departments. The first floor will become the chemical hall, the faculty room will be the dean's office, and the present dean's office will be given to the janitor. The old chemical laboratory has been converted into a faculty room. The main hall on the second floor will become the anatomical hall, the preparation room has been enlarged, and the old dissection room will become the museum hall. The annex to the main building has been widened and a third story added. The first and second floors will contain laboratories, and the third floor the dissecting room.

